# Patient-Reported Outcomes in Integrated Care: A Frontier of Opportunities and Challenges

**DOI:** 10.5334/ijic.9828

**Published:** 2025-08-22

**Authors:** Nicola Anderson, Sarah E. Hughes, Olalekan L. Aiyegbusi, Philip Collis, Robin Miller, Melanie Calvert

**Affiliations:** 1 Centre for Patient-reported Outcomes Research (CPROR), Person-centred Research, Department of Applied Health Sciences, College of Medicine and Health, University of Birmingham, UK; 2 National Institute of Health and Care Research (NIHR) Applied Research Collaboration (ARC) West Midlands, University of Birmingham, UK; 3 NIHR Biomedical Research Centre, University of Birmingham, UK; 4 University Hospitals Birmingham NHS Foundation Trust, Birmingham, UK; 5 NIHR Blood and Transplant Research Unit in Precision Cellular Therapeutics, University of Birmingham, UK; 6 Birmingham Health Partners Centre for Regulatory Science and Innovation, University of Birmingham, UK; 7 Patient Partner, CPROR, Institute of Applied Health Research, University of Birmingham, UK; 8 Department of Social Work and Social Care, School of Social Policy & Society, College of Social Sciences, University of Birmingham, UK

**Keywords:** patient-reported outcomes, electronic patient-reported outcome measures, patient-reported experience measures, quality of life, telemedicine, integrated care

## Abstract

Effective care integration requires the right information, at the right time, with care and support delivered in the right environment. Patient-reported outcome measures [PROMs] offer a mechanism to support collaborative integrated care to help people understand their conditions better, live well, and remain independent. Through the lens of the delivery of integrated care in England, we consider the opportunities and challenges associated with the use of PROMs across health and care.

## Introduction

Patient-reported outcomes offer important insights on the impact of illness, care, and treatment direct from an individual [1,2]. Initially designed to research the effectiveness and tolerability of treatments, such data are typically collected using methodologically robust self-report questionnaires, called patient-reported outcome measures [PROMs]. Increasingly, digital systems now collect PROMs and contribute to the delivery of patient-centred health care, value-based payments, and self-management approaches [3]. In social care settings measures such as the Adult Social Care Outcomes Toolkit (ASCOT) [4], designed for use with adults accessing services, unpaid and paid carers, and social care support settings, are used to support social care decision-making, research, and evaluation [5].

This commentary outlines the potential opportunities for PROMs use in integrated care [IC], highlights key challenges within this context, and enablers to optimise collection and use of this data. It used experiences within England to draw out learning for their application in other countries.

## Potential opportunities of PROMs within integrated care

Policy interest in the systematised use of PROMs in integrated care systems in England aligns with policy aspirations for more holistic approaches and a shift from supporting people when they get ill, to helping people stay well and as independent as possible [6,7]. As a result, the use of PROMs to assess the effectiveness of IC is increasing [8].

At an individual level, PROM data can be used to inform that person’s own care. In healthcare settings PROMs support individual-provider communication by prompting patients to reflect on their health, giving them permission to raise issues with clinicians [9]. Use of remote symptom monitoring systems based on PROMs may offer a solution to reduce outpatient waiting lists and tailor care to those in greatest need [10]. They can support person-centred shared decision-making by offering the individual perspective on symptoms, current care needs and goal setting [3]. Longitudinal PROM data supports conversations about care escalation and preferences in the event of declining health and increasing need.

At organisational and system levels, PROM data may be aggregated for use in benchmarking of performance metrics to support quality improvement. PROM data supplements other outcome data i.e., place of care, readmissions, mortality rates [11], to better understand the population challenges and overall effectiveness of IC initiatives [8].

Alongside PROM data, patient-reported experience measures [PREMs] assess services from the recipient’s viewpoint. Patient experience is multi-dimensional and includes relational [interactions], functional [result satisfaction] and integration aspects [12]. PREMs are usually completed anonymously, to enable patients to provide honest feedback [13]. Aggregated PREM data can indicate overall care quality [14].However, PREMs have been criticized for not effectively capturing what matters most to people at the service level, and for lacking timely and consistent measurement to support local quality improvement [15]. For example, in England, the NHS Friends and Family test [FFT] invites feedback on the overall experience of using the service. When combined with supplementary follow-up questions, the FFT provides a mechanism to identify services working well and those working poorly [16]. However, for July 2023, the national FFT response rates were only 22% for in-patients and 11% for accident and emergency department attendees [13].

## Potential challenges to PROMs within integrated care

The heterogeneous nature of IC settings, the diverse range of conditions, symptoms, social circumstances, and outcomes and the variations in models of care between sectors result in fragmented PROM selection and use [17]. There is often a disconnect between national policy and the local vision for IC which can lead to conflicting priorities. This is typically orientated around national focus on usage of acute medical care activity over community health and adult social care [18].

A scoping review of 216 PROMs deployed in social and integrated care settings found that while stakeholders viewed PROMs use as feasible and acceptable, major barriers existed including measure selection, administration, and data management [19]. Similarly, although there is evidence from stakeholders in England suggesting that PROMs and PREMs data are the best measures of effective integration, lack of agreement around measure selection and responsibility for measurement are major barriers [20].

In integrated healthcare settings, particularly oncology where PROMs are used to monitor disease symptoms which are linked to care-pathways, there is evidence of overall improved outcomes and cost-effectiveness [21]. However evidence to show the benefit of using PROMs and PREMs for benchmarking is lacking, often due to ineffective implementation strategies [22]. A recent systematic review identified PREMs in use across healthcare and only identified two measures focusing on care integration: they concluded that PREMs largely focus on a single episode of care, exhibiting large variability in both the number and type of validity and reliability testing [14].

An ongoing programme of research within England focused on the use of PROMs within IC has highlighted lack of expertise and awareness amongst professionals, patients, and carers to support their effective implementation [5,10,19]. Insufficient understanding of how PROMs are developed, validated and used will impact both purpose of collection and measure choice leading to measure variability in content, quality, and interpretability. There is also the risk of unvalidated surveys presented as PROMs.

A lack of motivation to complete by patients connected with professionals not explaining questionnaires [23] means that opportunities offered by PROMs and PREMs for individual care or service evaluation are unrealised. Research suggests responses are sometimes not reviewed or analysed and may be collected as a tick box exercise, with PROMs completion rates ranging from 50% to above 80% [24]. For longitudinal PROMs, the rate of completion significantly decreases over time. Missing data has huge implications and may not be random, with factors such as worsening health, inequitable access to health and care and the internet for remote monitoring limiting the use PRO data [24].

Evidence on PROMs use is largely derived from healthcare settings and may not be directly transferable to IC and describing these tools as ‘patient-reported’ outcome measures is implicitly healthcare focused. Arguably a shared common language is required to support uptake if we wish to capture self-reported outcome data across an individual’s journey involving different types of care. Describing these self-report questionnaires in terms of the construct being measured, such as symptom burden, quality of life or activities of daily living questionnaires may resonate better across services and those completing the measures.

## Goals of PROMs within integrated care

The goal is harmonised, flexible, and interoperable digital PROM collection systems which use validated, interpretable measurement tools that can be linked to cross organisational data sources for individual and aggregate use. PROM assessment must be inclusive and equitable [25]. PROMs integration must be grounded in emerging evidence of what works, when and in which contexts; with research undertaken in a variety of IC settings to understand contextual effects. Existing workforce strategies often focus on traditional models of care, ignoring the shifting of tasks in IC from practitioners to unpaid carers and communities. Without careful consideration of PROMs implementation there is a danger of introducing further problems and inequities. When choosing and implementing PROMs we need to consider language and cultural issues [25], together with the potential to cause respondent burden; particularly if people are asked to self-report on multiple outcomes using long or several questionnaires [26]. Table 1 sets out key enablers to support attainment of these goals.

**Table 1:** Enablers for implementing PROMs in integrated care settings.


**Harmonisation** 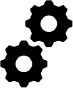	Shared outcomes at place level using tools such as the Shared outcomes toolkit for IC systems i.e., in Leeds, UK, PROM data for frailty populations is collected using the Patient-Reported Outcomes Measurement Information System [PROMIS] Global Health measure and a measure of person centred coordinated care to measure the outcome described as ‘Living and ageing well’ [27]. Flexible system design to ensure electronic health record compatibility and Interoperability to support data sharing and data linkage thorough common data standards and information governance frameworks [28] [29] Development of shared and common terminology Initiatives to share PROMs best practice

**Training** 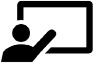	Develop and promote PROMs awareness and expertise using existing resources such as the PROTEUS-Practice guidelines [30] applicable to a broad range of environments, including integrated health systems Define and communicate key PROM system objectives, such as purpose of completion and who is accessing the data [31] Guidance on systemised approaches to review and action of PROM results and managing expectations.

**Resources** 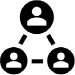	Harness existing electronic platforms to administer PROMs Development of dashboard approaches supporting PROMs data alerts, effective interpretability linked to tailored decision support and self-management advice. Use process mining techniques to understand existing care pathways and how PROMs might fit/impact [32] e.g., PROMs informed symptom management that aligns with existing workflow

**Inclusivity** 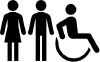	Involve all interested parties in design and implementation, focusing on local needs/priorities Identify specific needs of underserved populations i.e., health/digital literacy and access issues Optimise accessibility; allow onsite completion via kiosk/tablet, enable ‘bring your own device’ Investigate cultural applicability of chosen measures including language availability. Be aware of distrust in systems or providers, or impact of mental and physical health issues that may lower an individual’s commitment to complete PROMs [33], Developing methods for wider inclusion of people with severe intellectual, communication and cognitive impairments i.e., easy read versions/carer versions. Examples from ASCOT suite of tools in social care [34,35] New and extended roles for clinical ‘champions’ and ‘patient navigators’ to support initiatives for PROMs completion

**Burden reduction** 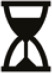	Ensure evaluation includes care service users to ensure PROM systems are not over-digitising the person’s experience or adding excessive burden. Utilise expert recommendations on how to reduce respondent burden associated with PROMs [26] Flexible data collection features offering efficient, easy user experience with completion reminders Invest in emerging technologies i.e. Computer Adaptive Testing and item banks to reduce questionnaire burden

**Future Research** 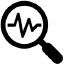	To investigate empirical evidence of benefit of using PROMs in IC Into measures that may offer most benefit within IC settings i.e., to inform care associated with multiple long-term conditions Employ PROM/PREM data within quality improvement initiatives using in-depth analysis and Plan-Do-Study-Act-cycles [22].


## Conclusion

PROMs offer a mechanism to support the delivery of person-centred integrated care, with opportunities to influence individual care and take a central role in assessing the overall effectiveness of integrated services to inform service design and improvement. However, significant challenges exist in optimising the use of PROMs and PREMs, necessitating a shift in cultural attitudes to place PROMs on par with other forms of data to support care planning and delivery, as well as finding solutions to system and technical barriers.
